# Characterization of the Complete Genome of *Arthrobacter globiformis* Cluster FF Bacteriophage QuinnAvery

**DOI:** 10.17912/micropub.biology.001994

**Published:** 2026-04-06

**Authors:** Elaine P Akst, Demelash Areda, Eric J Battaglioli, Christopher W Beck, Marina L Bogush, Steven M Caruso, Anne M Cedergren-Healy, Saroj V Chirravuri, Davida L Crossley, Katherine D'Amico-Willman, Veronique A Delesalle, Rebekah F Hare, Deborah L Harris, Jennifer R Ingram, Vedham Karpakakunjaram, Behnam Khatabi, Karen K Klyczek, Jonathan N Lawson, Cori Leonetti, Stephanie L Mathews, Matthew T McDonald, Katelyn M Mika, Ugonna C Morikwe, Zachary L Nikolakis, Xavier S Purroy, Amy B Ryan, Heather S Schiller, Mozhgan Sepehri, David E Starkey, Levi Storks, Kissaou T Tchedre, Ana C Vallor, Nic M Vega, Daniel E Velez-Ramirez, Emily E Whitaker, Daniel C Williams, Nicholas M Young, Christina H Zagorac, Deborah Jacobs-Sera, Denise Monti, Daniel A Russell, Bethany Wise, Marcie H Warner

**Affiliations:** 1 Biology Department, Montgomery College, Rockville, MD, US; 2 Department of Biology, Scottsdale Community College, Scottsdale, AZ, US; 3 Department of Biology, Emory University, Atlanta, GA, US; 4 Department of Biological and Biomedical Sciences, Rowan University, Glassboro, NJ, US; 5 Department of Biological Sciences, University of Maryland, Baltimore County, Baltimore, MD, US; 6 Department of Math and Science, GateWay Community College, Phoenix, AZ, US; 7 Department of Science and Mathematics, Mississippi University for Women, Columbus, MS, US; 8 Department of Biological Sciences, North Carolina State University, Raleigh, NC, US; 9 Department of Biology, Gettysburg College, Gettysburg, PA, US; 10 Department of Biology, Gonzaga University, Spokane, WA, US; 11 Department of Biology, Case Western Reserve University, Cleveland, OH, US; 12 Department of Biological Sciences, University of Pittsburgh at Greensburg, Greensburg, PA, US; 13 Department of Natural Sciences, University of Maryland Eastern Shore, Princess Anne, MD, US; 14 Department of Biology, University of Wisconsin–River Falls, River Falls, WI, US; 15 Department of Biology, Baylor University, Waco, TX, US; 16 Biology Department, Pima Community College, Tucson, AZ, US; 17 Department of Biology, Drexel University, Philadelphia, PA, US; 18 Department of Biological Science, University of Tulsa, Tulsa, OK, US; 19 Department of Biology, North Carolina A&T State University, Greensboro, NC, US; 20 Department of Biotechnology, Austin Community College, Austin, TX, US; 21 Department of Biological Sciences, Florida International University, Miami, FL, US; 22 Department of Biology, State University of New York Plattsburgh, Plattsburgh, NY, US; 23 Department of Biology, Marist University, Poughkeepsie, NY, US; 24 Department of Biology, University of the Incarnate Word, San Antonio, TX, US; 25 Department of Biology, University of Detroit Mercy, Detroit, MI, US; 26 Department of Microbiology, Immunology, and Molecular Genetics, University of California, Los Angeles, Los Angeles, CA, US; 27 Department of Molecular & Biomedical Sciences, University of Maine, Orono, Maine, US; 28 Department of Biology, Coastal Carolina University, Conway, SC, US; 29 Department of Biology, Health, and Environment, The University of Texas at San Antonio, San Antonio, TX, US; 30 Department of Biological Sciences, University of Pittsburgh, Pittsburgh, PA, US; 31 Science Education Alliance, Howard Hughes Medical Institute, Chevy Chase, MD, US

## Abstract

Phage QuinnAvery produces turbid plaques in *Arthrobacter globiformis* B-2979 lawns, displays a siphovirus morphology, and is clustered with FF actinobacteriophages. The 43,177 bp genome of QuinnAvery is predicted to contain a tRNA-Arg(TCT) gene and 69 protein-coding genes. Homologs of a subset of QuinnAvery’s predicted genes have putative roles in viral structure, particle assembly, host cell lysis, and lysogeny. QuinnAvery’s genome is predicted to contain two tyrosine integrases, similar to many other cluster FF phages. However, the area immediately surrounding the integration cassette in QuinnAvery is distinct from all other annotated members of cluster FF.

**Figure 1. Phage QuinnAvery morphological and genomic characteristics:** (A) QuinnAvery produces turbid plaques in an *A. globiformis* B-2979 lawn embedded in an 0.4% PYCa agar overlay after incubation at 30 ^o^ C for 3 days. Plaques average 0.73 millimeters (+/- 0.16; n=40; image is magnified 2-fold). (B) QuinnAvery virion particle morphology was elucidated by transmission electron microscopy of particles stained with 1% wt/vol uranyl acetate. Images were captured using a Hitachi HT7800 at 80k magnification. (C) Phamerator map depicting predicted size, position, and function of 70 genes in the QuinnAvery genome. (D) Lysogens harboring QuinnAvery prophage elements are generated in approximately 53% of host cells. An equal sample of *A. globiformis* B-2979 was spread on a PYCa plate (left) and a PYCa plate that had been seeded with 10 ^8^ QuinnAvery plaque forming units (right) and incubated for 3 days at 30 ^o^ C. Plates show an efficiency of plating of approximately 53%. (E) Supernatant was collected from liquid cultures of two QuinnAvery lysogen candidates (1 and 2), serially diluted, and plated on a lawn of *A. globiformis* B-2979 and incubated at 30 ^o^ C for 1 day to assay for phage release. Controls (+ = QuinnAvery lysate; - = phage buffer) were also serially diluted and plated in parallel.

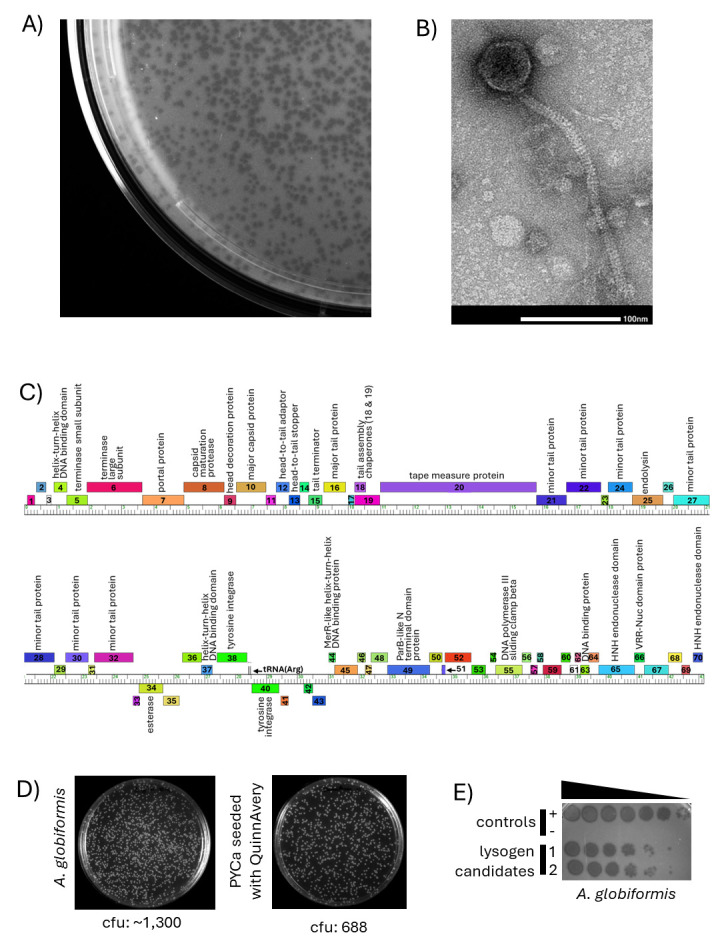



## Description

The global diversity of bacteriophages is exceptionally large and diverse (Hendrix et al., 1999; Pedulla et al., 2003; Suttle et al., 2005). Continuous identification and characterization of novel phages has significantly enhanced our understanding of viral function and evolution (Hendrix et al., 1999; Mavrich and Hatfull, 2017; Hatfull, 2022). To contribute to ongoing efforts to explore bacteriophage diversity, we report here the discovery and initial characterization of QuinnAvery, an *Arthrobacter globiformis* phage.

Phage QuinnAvery was discovered utilizing SEA-PHAGES protocols (Zorawik et al., 2024). Briefly, an environmental soil sample was collected in Linthicum Heights, Maryland, USA (39.2015 N, 76.68484 W) in June 2024. Soil was agitated in peptone-yeast extract-calcium (PYCa) liquid medium in the presence of *A. globiformis* B-2979 at 30 ^o^ C for 24 hours. Following centrifugation, the supernatant was passed through a 0.22 mm polycarbonate filter to remove residual soil particulates and bacterial cells. A fraction of the collected filtrate was mixed with 0.4% top agar and *A. globiformis* and overlaid onto a solid PYCa plate. Turbid plaques measuring 0.73 millimeters (+/- 0.16; n=40) were present following incubation at 30 ^o^ C for 1 day ( Figure 1A ). Well-isolated plaques were utilized for three rounds of purification prior to generation of a high titer liquid lysate. Transmission electron microscopy was performed on a sample of lysate that had been negatively stained with 1% uranyl acetate. This analysis revealed that phage QuinnAvery displays siphovirus morphology with a capsid diameter of approximately 51.5 nm (+/- 0.25 nm) in diameter and a tail length of approximately 258 nm (+/- 1 nm) (n=2; Figure 1B ).

Genomic DNA was extracted from a sample of high titer lysate utilizing the Promega Wizard DNA Clean-Up Kit, sequencing libraries were created with the Illumina XLEAP-P1 kit, and DNA sequencing was performed on an Illumina NextSeq 1000 to approximately 5,933-fold coverage. Approximately 4,000,000 raw &nbsp;single-end 150-bp reads were trimmed with cutadapt 4.7 (using the option: –nextseq-trim 30) and filtered with skewer 0.2.2 (using the options: -q 20 -Q 30 -n -l 50) prior to assembly and finished using Unicycler 0.5.1 (Wick et al., 2017) and Consed v.29 (Gordon et al., 2013) with default parameters. These analyses determined that the genome of Quinn Avery is 43,177 bp in length, is composed of 64.9% GC nucleotide pairs, and terminates in a 3’ single-stranded overhang (5’-TCCGCCGCGTGA-3’). Using the Gene Content tool on PhagesDB (Russell and Hatfull, 2017), QuinnAvery shares between 71% to 79% gene content similarity with annotated cluster FF phages, including Elesar, Cole, Guasanita, Nandita, Popper, Ryan, and Zaheer. This degree of similarity exceeds the threshold value of 35% gene content similarity (Pope et al., 2017b) necessary for QuinnAvery to be assigned to actinobacteriophage Cluster FF.

Initial auto-annotation was performed using Glimmer v3.02 (Delcher et al., 1999) and GeneMark v2.5 (Besemer & Borodovsky, 2005) and manually refined using Phamerator v580 (Cresawn et al., 2011), DNA Master v5.23.6 (Pope and Jacobs-Sera, 2018), Starterator v587 (github.com/SEA-PHAGES/starterator), and PECAAN (discover.kbrinsgd.org). Functional assignments were evaluated using BLASTp v 2.2.26 (using NCBI nonredundant database and the database at phagesdb.org; Altschul et al., 1990) and HHpred (using PDB_mmCIF70, PfamA, and NCBI Conserved Domain databases; Söding et al., 2005). A single tRNA ^ARG^ gene was identified by Aragorn v1.2.38 (Laslett & Canback, 2004) and tRNAscan-SE v2.0 (Lowe & Chan, 2016). The presence of transmembrane helices was evaluated with Deep TMMHM v1.0.42 (Hallgren et al., 2022). Default parameters were utilized for all programs other than DNA Master, which was set according to instructions in the SEA-PHAGES bioinformatics guide (Pope, Jacobs-Sera et al., 2017).

The finalized annotation includes a total of 69 predicted protein-coding genes, of which 39 were assigned putative functions ( Figure 1C ). The first thirty-two predicted genes in the left arm of the chromosome are oriented in the forward direction and contain an abundance of genes predicted to encode structural and assembly proteins. Multiple genes with predicted roles in nucleic acid processing and metabolism are located in the final 27 forward-oriented genes at the right end of the genome. Genes with predicted roles in lysogeny are centrally located in an area of the genome where gene orientation switches twice from reverse to forward. Of the eight annotated cluster FF phages, all but Elesar contain two predicted tyrosine integrases. The identification of multiple candidate integrases in a phage genome is rare (Hatfull et al., 2010; Klyczek et al., 2017), though it has been observed previously in a small number of *Arthrobacter* and *Gordonia* phages (Pope, Mavrich and Hatfull, 2017). Although lysogen candidates harboring QuinnAvery prophage have been isolated (Figures 1D and 1E), additional studies are necessary to confirm whether both integrases (genes 38 and 40), which share only 44% amino acid similarity, are functional (Wise and Sivanathan, 2025).

All previously annotated cluster FF phages with two predicted integrases contain at least one predicted gene between the integrases in addition to the tRNA ^ARG^ ; the identity and orientation of these genes is variable. QuinnAvery is unusual in that there are no predicted protein-coding genes between its predicted integrases; these genes are separated only by the tRNA ^ARG^ gene. QuinnAvery is also unique among cluster FF phages in that it contains two forward genes positioned upstream of the first predicted integrase; gene 36 has no recognizable functional domains, but gene 37 is predicted to contain a helix-turn-helix motif. Elucidation of the functions of these proteins may yield interesting insights into the complexities of establishing and maintaining lysogeny.

&nbsp;

**Nucleotide sequence accession numbers**

QuinnAvery is available at GenBank with Accession No. PQ844483 and Sequence Read Archive (SRA) No. SRX27124803.
